# Anisotropic Strange Star in 5*D* Einstein-Gauss-Bonnet Gravity

**DOI:** 10.3390/e23081015

**Published:** 2021-08-06

**Authors:** Mahmood Khalid Jasim, Sunil Kumar Maurya, Ksh. Newton Singh, Riju Nag

**Affiliations:** 1 Department of Mathematical and Physical Sciences, College of Arts and Sciences, University of Nizwa, P.O. Box 33, Nizwa PC 616, Oman; mahmoodkhalid@unizwa.edu.om (M.K.J.); rijunag@gmail.com (R.N.); 2 Department of Physics, National Defence Academy, Khadakwasla, Pune 411023, India; ntnphy@gmail.com

**Keywords:** anisotropy, EGD gravity, equation of state (EOS), strange star

## Abstract

In this paper, we investigated a new anisotropic solution for the strange star model in the context of 5D Einstein-Gauss-Bonnet (EGB) gravity. For this purpose, we used a linear equation of state (EOS), in particular $p_{r}=\beta \;\rho +\gamma$, (where β and γ are constants) together with a well-behaved ansatz for gravitational potential, corresponding to a radial component of spacetime. In this way, we found the other gravitational potential as well as main thermodynamical variables, such as pressures (both radial and tangential) with energy density. The constant parameters of the anisotropic solution were obtained by matching a well-known Boulware-Deser solution at the boundary. The physical viability of the strange star model was also tested in order to describe the realistic models. Moreover, we studied the hydrostatic equilibrium of the stellar system by using a modified TOV equation and the dynamical stability through the critical value of the radial adiabatic index. The mass-radius relationship was also established for determining the compactness and surface redshift of the model, which increases with the Gauss-Bonnet coupling constant α but does not cross the Buchdahal limit.

## 1. Introduction

In the last few decades, the study of various compact stars (neutron stars, white dwarfs, black holes, dark stars, gravastars, etc.) has been an active topic of research. Compact stars are objects with high density or a very high magnitude of mass within a small radius. Barring black holes, all other kinds of compact stars are referred to as degenerate stars. Until now, the exact nature of compact stars was unknown to us, but it is known that the formation of compact stars signifies the endpoint of stellar evolution. The theoretical modeling of various physical features of compact stars has fascinated researchers, as they provide great testing conditions for hugely compact matter content in extreme conditions. In a few past works, various massive objects, such as black holes, pulsars, white dwarfs, neutron stars, etc., were mathematically modeled in the framework of the general theory of relativity [1]. Delgaty and Lake [2] conducted a detailed analysis to obtain various analytical solutions, describing realistic stellar objects. We must also mention the pioneering research of Tolman [3], Oppenheimer and Volkoff [4], Schwarzschild [5] and Chandrasekhar [6] for the development of theoretical models of stellar objects. Interior solutions of a star with uniform density were obtained by Schwarzschild [5]; new solutions considering static spheres of fluid were obtained by Tolman [3]; and Oppenheimer and Volkoff [4] used the equation of state for a cold Fermi gas, with the consideration of general relativity, and analyzed the gravitational equilibrium of neutron masses. Chandrasekhar [6] considered relativistic effects and produced new models for white dwarfs. To study the stability of the fluid spheres, some work was also extended to higher dimensions [7].

In recent years, modified theories of gravity have gained much attention among researchers. The fact that modified theories of gravity can play a key role in unraveling such mysteries as the accelerated expansion of the universe makes them even more fascinating for researchers. The mathematical rationale behind them is that the non-zero contribution to the dynamics is yielded by higher-order derivative curvature terms.

Particularly, the Einstein–Gauss-Bonnet (EGB) theory is very promising in this context. In fact, in string theory, the Einstein-Gauss-Bonnet gravity appears automatically when considering the effective action in a low energy limit. Mathematically, it is a generalized form of Einstein gravity, with the addition of an extra term along with the Einstein–Hilbert (E-H) action. If one varies this extra term with respect to the metric only, a system of second-order equations can be generated, and that is the reason that this theory keeps many properties intact of classical general relativity. It must be mentioned that in 4D, both EGB and the general theory of relativity are equivalent, while EGB does indicate new results in the context of gravitational collapse. Additionally, for the inhomogeneous spherically symmetric distribution of dust, the study of the causal structure of singularities differs from that of general relativity [8]. Using EGB theory, several black hole models have been studied. For example, Myers and Simons [9] and Torii and Maeda [10] studied black hole solutions, using EGB theory. Higher-dimensional solutions of Einstein theory were generalized by Boulware and Deser [11], due to Tangherlini [12] and Myers and Perry [13] including the EGB theory by considering the quadratic curvature terms. Maeda [14] studied the inhomogeneous collapse of non-interacting, pressure-free particles or dust. Jhingan and Ghosh [8] obtained the exact solutions. Dadhich et al. [15] proved that the Schwarzschild interior solution for constant density remains the same for higher-dimensional Einstein theory and EGB gravity. Exact solutions for static, spherical perfect fluid were obtained by Hansraj et al. [16], while a comparison was conducted between Einstein gravity and Einstein-Gauss-Bonnet gravity by Bhar et al. [17] in Krori Barua spacetime. Recently, Malaver and Kasmaei [18] obtained the strange star model in 5D EGB gravity under linear and non-linear EOS of the form $p_{r}\propto \rho^{n}$, where n=1,2.

On the other hand, Martino et al. [19] took the cosmographic approach and studied the possibility of F(R,G) gravity being able to lead the wide spectral range spanning from ultraviolet to infrared. Alimohammadi and Ghalee [20] considered the quantum effects of the deceleration to acceleration transition in the context of modified F(R,G) gravity and showed that in cases where there is no transition in the classical level, the transition can be induced by quantum effects. Böhmer and Lobo [21] studied the stability of the Einstein static universe by considering the perturbations in the form of modified Gauss-Bonnet f(G) gravity. Cognola et al. [22] reconstructed the scalar Gauss-Bonnet and modified Gauss-Bonnet gravities on the basis of the universe expansion history. A scalar field model having non-minimal kinetic couplings along with additional coupling to the four-dimensional GB invariant was the approach taken by Granda [23], and two solutions with the dynamical equation of state were considered: the first solution was able to describe the early time power-law behavior and the second solution was able to describe the phantom phase of the universe, which can lead to dark energy depending on suitable parameters. Ivanov and Toporensky [24] considered both *R* and *G* (*G* being Gauss-Bonnet invariant) corrections to the Einstein gravity, and they studied specifically the case for which both these terms become equally important while describing power-law solutions. Li et al. [25] tried to explain the late-time accelerated expansion of the universe by adding the Gauss-Bonnet term *G* to the gravitational action, and showed that the f(G) models are very much constrained by cosmological data. Nojiri and Odintsov [26] took into account dark energy by adding the Gauss-Bonnet term in the Einstein action and showed that their model can pass solar system tests, and also it can describe the late time cosmology. In this connection, Nojiri et al. [27] proposed a dark energy model influenced by string/M theory and based on the Gauss-Bonnet modified theory of gravity. They took into account the additional coupling with the Gauss-Bonnet invariant with the standard gravity scalar and showed that the phantom phase of the late time universe can be described by this term when the scalar is canonical. Recently, Astashenok et al. [28] considered f(R) gravity and showed that the modified theories of gravity can be distinguished from the standard GR as a result of the gravitational redshift of the thermal spectrum that emerges from the surface of the star. The GW19081W event was studied by Astashenok et al. [29] in the context of f(R) gravity; they showed that the secondary component of the compact binary of GW19081W cannot be a strange star, but can be anything in the form of a black hole, a neutron star or a rapidly rotating neutron star. Odintsov et al. [30] studied the EGB theories; by fulfilling the condition that the EGB theory has to be consistent with the GW19081W event, they presented their model, which is consistent with observable data.

On the other hand, under the influence of extreme matter density, the pressure waves of a compact object are split into two components, that is, the radial pressure ($p_{r}$) and the tangential pressure ($p_{t}$). This division of pressure components results in anisotropy inside the compact object and is measured by the quantity $\Delta =p_{t}-p_{r}$. Positive anisotropy means the radial force acting outwards that helps to prevent gravitational collapse. The role of anisotropy in relativistic spheres and critical aspects of anisotopic pressure together with the equation of hydrostatic equilibrium for local anisotropy were studied by Bowers and Liang [31], while the cause–effect of local anisotropy in self-gravitating spheres were explained by Herrera and Santos [32]. Static interior solutions for relativistic and anisotropic matter distributions were obtained by Harko and Mak [33]. Some significant facts responsible for this splitting of pressure components leading to anisotropy include the existence of a solid core, pion condensation, and electric field [34], and the existence of type 3A superfluid [35], magnetic field, phase transitions, to name a few. Additionally, while studying the relativistic spheres of fluid, the context of anisotropy plays a significant role [36,37,38,39,40,41,42,43,44,45,46,47]. Bhar et al. [48] took the Tolman VII form of gravitational potential, with a linear combination between energy density and radial pressure, and studied the relativistic objects having locally anisotropic matter content. Solutions for the Einstein–Maxwell field equations with charged and symmetric spacetime, having anisotropic pressure, were studied by Sunzu et al. [49], Feroze and Siddiqui [50,51] and Malaver [52,53].

In the current article, we considered 5*D* Einstein-Gauss-Bonnet (EGB) gravity, and tried to find an anisotropic solution for a strange star model. In order to do this, we have chosen a well-behaved ansatz for gravitational potential $g_{rr}$ and a linear EOS $p_{r}=\beta \rho +\gamma$ (β and γ are constants). Thus, we found the other gravitational potential along with the key thermodynamic variables, such as radial, tangential pressure, and energy density. We then found the constant parameters by using the matching condition technique, while matching the much known Boulware-Deser solution at the boundary. We tested how the model is physically acceptable. In addition, we used the modified TOV equation in order to test the hydrostatic equilibrium. The dynamical stability was studied through the critical value of the radial adiabatic index.

The present article is divided into six sections. The first one of them is the introduction. In Section 2, we obtain the basic field equation for Einstein-Gauss-Bonnet (EGB) gravity. In Section 3, we shed light on the anisotropic solution for the star model in 5*D* Einstein-Gauss-Bonnet gravity, while Section 4 is about the boundary conditions where we use the exterior vacuum solution proposed by Boulware-Deser. This section is divided into two subsections: in Section 5.1, we discuss regularity conditions; in Section 5.2, the causality is studied; in Section 5.3, the stability of anisotropic compact objects via cracking is discussed; in Section 5.4, the stability criterion and the adiabatic indices are studied; while in Section 5.5, the hydrostatic equilibrium via modified TOV equation is analyzed. The mass-radius relationship and the M−R curves are discussed in Section 5.6 and Section 5.7, respectively, to determine the compactness and maximum mass limit. The last section is based on the concluding remarks, where we summarize all the findings and aspects of this current study. However, the complexity for self-gravitating fluid distributions under the static spherically symmetric spacetime is discussed in the Appendix A.

Throughout the discussion, we choose the sign conventions (−,+,+,+), together with universal constants G=c=1.

## 2. Basic Field Equations for Einstein-Gauss-Bonnet (EGB) Gravity

Let us write the action for EGB gravity with matter field in *D*-dimensional spacetime, which is given by the following:

(1) $$\begin{array}{c} I_{G}=\frac{1}{16\pi }\int d^{D}x\sqrt{-g}\left[R+\alpha L_{\mathrm{GB}}\right]+S_{\mathrm{matter}}, \end{array}$$

where *R* and *g* are the *D*-dimensional Ricci scalar and the determinant of the metric tensor $g_{ij}$, respectively. Here, $L_{\mathrm{GB}}$ denotes a Lagrangian for the Gauss-Bonnet term, while $S_{\mathrm{matter}}$ is the action of the matter field. However, the constant α is defined as a Gauss-Bonnet coupling constant with the dimension [length]${}^{2}$. Now, the term $L_{\mathrm{GB}}$ can be defined as the following:

(2) $$L_{\mathrm{GB}}=R^{ijkl}R_{ijkl}-4R^{ij}R_{ij}+R^{2},$$

where $R_{ijkl}$ and $R_{ij}$ denote the Riemann curvature tensor and Ricci tensor, respectively, while *R* is the Ricci scalar. In EGB theory, it is necessary to consider the coupling constant α to be a positive definite for achieving the stability of Minkowski spacetime [54,55]. Additionally, the coupling constant α links with the inverse string tension and is considered to be a positive value in string theory [11] with dimension of [length]${}^{2}$. However, some authors chose both cases of α>0 and α<0 (see Refs. [56,57] for further discussion). So, in our work, we consider α≥0. On the other hand, we mention here that the present form of this action is known also to track from the low energy limit of heterotic superstring theory.

Now, we obtain the equation of motion by varying the action (1) with respect to the metric $g^{ij}$ as the following:

(3) $$8\pi T_{ij}=G_{ij}+\alpha \;H_{ij},$$

where $T_{ij}$ is the energy–momentum tensor for the matter field. However $G_{ij}$ and $H_{ij}$ are called the Einstein tensor and Gauss-Bonnet term, respectively, which can be given by the following expressions:

(4) $$\begin{array}{c} G_{ij}=R_{ij}-\frac{1}{2}R\;g_{ij},\;\;\mathrm{and}\;\;H_{ij}=2\left(RR_{ij}-2R_{ik}R_{j}^{k}-2R_{ijkl}R^{kl}-R_{ikl\delta }R_{j}^{kl\delta }\right)-\frac{1}{2}\;g_{ij}\;L_{\mathrm{GB}}. \end{array}$$

Here, we derived the energy tensor $T_{ij}$ from $S_{\mathrm{matter}}$ in the action (1) as the following:

(5) $$\begin{array}{c} T_{ij}=-\frac{2}{\sqrt{-g}}\frac{\delta \left(\sqrt{-g}S_{m}\right)}{\delta g^{ij}}. \end{array}$$

We would like to mention here that there is no contribution of the GB term in the field equations when D≤4, i.e., $H_{ij}\equiv 0$. Here, we are interested in finding the static spherically symmetric anisotropic solutions of Equation (3). Therefore, we consider a static and spherically symmetric metric line element in five-dimensional form as follows:

(6) $$\begin{array}{c} ds_{5}^{2}=-e^{2\nu (r)}dt^{2}+e^{2\lambda (r)}dr^{2}+r^{2}\left(d\theta^{2}+\sin^{2}\theta \;d\phi^{2}+\sin^{2}\theta \sin^{2}\phi \;d\psi^{2}\right), \end{array}$$

where ν(r) and λ(r) are functions of radial coordinate *r*. Since we consider the matter distribution as anisotropic, the energy–momentum tensor $T_{ij}$ for an anisotropic fluid can be given as the following:

(7) $$\begin{array}{c} T_{ij}=\left(\rho +p_{t}\right)u_{i}u_{j}+p_{t}g_{ij}+\left(p_{r}-p_{t}\right)\chi_{i}\;\chi_{j}, \end{array}$$

where ρ(r) is the energy density of matter, while $p_{r}\left(r\right)$ and $p_{t}\left(r\right)$ are the radial and tangential pressures, respectively. We denote $u^{i}=e^{-\nu }\;\delta_{1}^{i}$ as a four velocity vector satisfying $u_{i}u^{i}=-1$ and $u_{i}\nabla^{j}u_{i}=0$. However, $\chi^{i}=e^{-\lambda }\;\delta_{1}^{i}$ is called the unit space-like vector, which is orthogonal to four-velocity vector $u^{i}$ satisfying the relation $\chi^{i}\chi_{i}=1$. On the other hand, we can easily verify by applying the Bianchi identity that the Einstein tensor ($G_{ij}$) and Gauss-Bonnet tensor ($H_{ij}$) are individually conserved, which shows that the energy–momentum tensor $T_{ij}$ of the matter field is conserved [58], i.e.,

(8) $$\begin{array}{c} \nabla^{i}\;T_{ij}=0\;⟹\;\nu^{\prime }\left(\rho +p_{r}\right)+p_{r}^{\prime }+\frac{3}{r}\left(p_{r}-p_{t}\right)=0.\; \end{array}$$

The above conservation Equation (8) is known as the Tolman-Oppenheimer-Volkoff equation in 5D EGB gravity. Now, the explicit equations for field Equation (3) under spherically symmetric metric (6) in EGB gravity can be written as the following:

(9) $$\begin{array}{ccc} & & \;8\pi \rho =-\frac{3}{e^{4\lambda }r^{3}}\left(4\alpha \lambda^{\prime }+re^{2\lambda }-re^{4\lambda }-r^{2}e^{2\lambda }\lambda^{\prime }-4\alpha e^{2\lambda }\lambda^{\prime }\right),\;\;\;\; \end{array}$$

(10) $$\begin{array}{ccc} & & \;8\pi p_{r}=\frac{3}{e^{4\lambda }r^{3}}\left\{-re^{4\lambda }+\left(r^{2}\nu^{\prime }+r+4\alpha \nu^{\prime }\right)e^{2\lambda }-4\alpha \nu^{\prime }\right\},\;\; \end{array}$$

(11) $$\begin{array}{ccc} & & \;8\pi p_{t}=\frac{1}{e^{4\lambda }r^{2}}\left(-e^{4\lambda }-4\alpha \nu^{\prime \prime }+12\alpha \nu^{\prime }\lambda^{\prime }-4\alpha \nu^{\prime 2}\right)+\frac{1}{e^{2\lambda }r^{2}}\{1-r^{2}\nu^{\prime }\lambda^{\prime }+2r\nu^{\prime }\\ & & \;-2r\lambda^{\prime }+r^{2}\nu^{\prime 2}\}+\frac{1}{e^{2\lambda }r^{2}}\left(r^{2}\nu^{\prime \prime }-4\alpha \nu^{\prime }\lambda^{\prime }+4\alpha \nu^{\prime 2}+4\alpha \nu^{\prime \prime }\right), \end{array}$$

Here “prime” denotes the differentiation with respect to *r*, only. Moreover, the gravitational mass function m(r) in 5D-EGB gravity can be calculated by the following formula (see ref. [59] for more details):

(12) $$\begin{array}{c} m\left(r\right)=\frac{8\pi }{3}\;\int_{0}^{r}\rho \left(x\right)\;x^{3}\;dx. \end{array}$$

Now, we aim to solve the above three field equations, which contain five unknown functions, namely, two metric functions λ(r) and ν(r), and three matter variables ρ(r), $p_{r}\left(r\right)$ and $p_{t}\left(r\right)$. Then, we need to specify two conditions to achieve consistency of the system, which is discussed in the next section.

## 3. Anisotropic Solution for Strange Star Model in 5*D* Einstein-Gauss-Bonnet Gravity

In this section, we will discuss the anisotropic solution in 5D Einstein-Gauss-Bonnet (EGB) gravity. Recently, some pinioning works on anisotropy on the compact star models have been widely discussed in classical general relativity and modified gravity theory by several authors [60,61,62,63,64,65,66,67,68,69,70,71,72,73,74]. Here, our aim is to generate an exact solution of the system of Equations (9)–(11) for anisotropic matter distribution. As earlier mentioned, field Equations (9)–(11) contain five unknowns, λ, ν ρ, $p_{r}$, and $p_{t}$, which require two conditions in order to solve the system exactly. Therefore, we use the two physically viable conditions: (i) linear equation of state (EOS), and (ii) a well-behaved ansatz, corresponding to $g_{rr}$ components of the metric function. These conditions are given as the following:

(13) $$\begin{array}{ccc} & & p_{r}=\beta \;\rho +\gamma ,\;\;\; \end{array}$$

(14) $$\begin{array}{ccc} & & \lambda =\frac{1}{2}\;\ln\left(1+br^{2}+cr^{4}\right). \end{array}$$

Here, β and γ are constants. We would like to mention here that the above linear EOS can also describe the MIT EOS for strange matter when $\beta =\frac{1}{3}$ and $\gamma =-\frac{4}{3}\;B$, where B is the Bag constant. Moreover, *b* and *c* are also positive constants with dimension $length^{-2}$ and $length^{-4}$, respectively. This choice of the metric function λ is well motivated and wildly used by several authors in other gravity, such as f(R,T) gravity, to find realistic compact star models [75,76]. Now, using Equations (9) and (10) together with EoS (13), we arrive at the following differential equations:

(15) $$\begin{array}{c} 12\alpha \left(e^{-2\lambda }-1\right)\left(-2\lambda^{\prime }\;\beta \;e^{-2\lambda }+2\nu^{\prime }\;e^{-2\lambda }\right)+r[-6+6e^{-2\lambda }+6\nu^{\prime }\;e^{-2\lambda }r-2\gamma r^{2}+3\beta (-2\\ +2e^{-2\lambda }+-2\lambda^{\prime }\;e^{-2\lambda }r)]=0. \end{array}$$

Now, using the condition (14), one can obtain a physically relevant solution for ν of the following form:

(16) $$\begin{array}{ccc} & & \;\nu =\frac{1}{3}[\nu_{3}\;r^{2}+\nu_{4}\;r^{4}+\frac{1}{6}c\;\gamma \;r^{6}+\frac{\alpha \left(\nu_{1}+\nu_{2}\right)\left(-3-3\beta +4\alpha \gamma \right)\ln\left[b+4\alpha \;c-\nu_{5}+2cr^{2}\right]}{\nu_{5}}\\ & & \;+\frac{3}{2}\;\beta \;\ln\left(1+br^{2}+cr^{4}\right)]+\ln C, \end{array}$$

Here, *C* is a dimensionless arbitrary constant of integration:

$$\begin{array}{ccc} & & \nu_{1}=b^{2}+b\left\{-8\alpha c+\sqrt{b^{2}-8\alpha bc+4c\left(-1+4\alpha^{2}c\right)}\right\}+2c[-1+8\alpha^{2}c\\ & & \;-2\alpha \sqrt{b^{2}-8\alpha bc+4c\left(-1+4\alpha^{2}c\right)}],\\ & & \nu_{2}=-b^{2}+b\left[8\alpha c+\sqrt{b^{2}-8\alpha bc+4c\left(-1+4\alpha^{2}c\right)}\right]-2c[-1+8\alpha^{2}c\\ & & \;+2\alpha \sqrt{b^{2}-8\alpha bc+4c\left(-1+4\alpha^{2}c\right)}],\\ & & \nu_{3}=\frac{1}{2}\left[\gamma -12\alpha c\left(1+\beta \right)+16\alpha^{2}c\gamma +b\left(3+3\beta -4\;\alpha \;\gamma \right)\right],\\ & & \nu_{4}=\frac{1}{4}\left[b\;\gamma +c\left(3+3\beta -4\alpha \;\gamma \right)\right],\\ & & \nu_{5}=\sqrt{b^{2}-8\alpha bc+4c\left(-1+4\alpha^{2}c\right)}. \end{array}$$

Now, we obtain the expressions for ρ, $p_{r}$ and $p_{t}$ by substituting Equations (13) and (14) into Equations (9)–(11) as follows:

(17) $$\begin{array}{ccc} & & \;8\pi \rho =\frac{3}{{\left(1+br^{2}+cr^{4}\right)}^{3}}[b^{3}r^{4}+cr^{2}\left(3+8\alpha cr^{2}+4cr^{4}+c^{2}r^{8}\right)+b(2+12\alpha cr^{2}+7cr^{4} \end{array}$$

(18) $$\begin{array}{ccc} & & \;+3c^{2}r^{8})+b^{2}\left\{4\alpha +3\left(r^{2}+cr^{6}\right)\right\}],\;\;\;\;\;\;\\ & & \;8\pi p_{r}=\frac{1}{{\left(1+br^{2}+cr^{4}\right)}^{3}}[\gamma {\left(1+cr^{4}\right)}^{3}+b^{3}\left(3\beta r^{4}+\gamma r^{6}\right)+3\;c\;\beta \;r^{2}\;(3+8\alpha cr^{2}+4cr^{4}\\ & & \;+c^{2}r^{8})+3b^{2}\left\{4\alpha \;\beta +r^{2}\left(3\beta +\gamma r^{2}\right)\left(1+cr^{4}\right)\right\}+3b\{\gamma {\left(r+cr^{5}\right)}^{2}+\beta (2+12\alpha \;cr^{2}\\ & & \;+7cr^{4}+3c^{2}r^{8})\}], \end{array}$$

(19) $$\begin{array}{ccc} & & \;8\pi p_{t}=\frac{1}{9\;{\left(1+br^{2}+cr^{4}\right)}^{4}\left\{1+br^{2}+cr^{4}+4a\left(b+cr^{2}\right)\right\}}\left[p_{t1}+p_{t2}\right]. \end{array}$$

Since we have specified all the unknown metric functions and matter variables, it is necessary to match the interior spacetime with a suitable exterior spacetime in order to find the arbitrary constants and free parameters involved in the solution.

## 4. Boundary Conditions

In this section, we explain the necessary boundary conditions for matching the interior solution with an appropriate static and spherically symmetric exterior vacuum solution. Since we are studying the interior anisotropic solution in 5D EGD gravity, the most suitable exterior vacuum solution is proposed by Boulware–Deser [11], which can be given by following line element:

(20) $$\begin{array}{c} ds_{5}^{2}=-\left[1+\frac{r^{2}}{4\alpha }\left(1-\sqrt{1+\frac{16\alpha M}{r^{4}}}\right)\right]dt^{2}+{\left[1+\frac{r^{2}}{4\alpha }\left(1-\sqrt{1+\frac{16\alpha M}{r^{4}}}\right)\right]}^{-1}dr^{2}\\ +r^{2}\left(d\theta^{2}+\sin^{2}\theta \;d\phi^{2}+\sin^{2}\theta \sin^{2}\phi \;d\psi^{2}\right). \end{array}$$

where *M* describes the total mass of the object at r=R, which is associated with the mass m(r) at boundary, i.e., M=m(R). It is highlighted that when coupling constant α→0, then exterior metric (20) converts into the 5D Schwarzschild solution. Now, we join smoothly the interior metric (6) with the Boulware–Deser [11] exterior metric at the boundary surface r=R. The smooth joining of the spacetimes states the continuity of the first fundamental across the boundary Σ, which can be given by $g_{tt}^{-}=g_{tt}^{+}$ and $g_{rr}^{-}=g_{rr}^{+}$. This condition yields the following:

(21) $$\begin{array}{c} e^{2\lambda^{-}}{|}_{r=R}=e^{2\lambda^{+}}{|}_{r=R}\;\mathrm{and}\;e^{2\nu^{-}}{{|}_{r=R}=e^{2\nu^{+}}|}_{r=R},\;\; \end{array}$$

Using Equations (6), (20) and (21), we obtain the following:

(22) $$\begin{array}{ccc} & & e^{2\nu (R)}=\left[1+\frac{R^{2}}{4\;\alpha }\left(1-\sqrt{1+\frac{16\;\alpha M}{R^{4}}}\right)\right], \end{array}$$

(23) $$\begin{array}{ccc} & & e^{-2\lambda (R)}=\left[1+\frac{R^{2}}{4\alpha }\left(1-\sqrt{1+\frac{16\;\alpha M}{R^{4}}}\right)\right]. \end{array}$$

On the other hand, the continuity of the second fundamental form also requires stellar objects, which leads to the vanishing of radial pressure at the boundary. This second fundamental gives the following:

(24) $$\begin{array}{c} {\left[\left(G_{ij}+\alpha \;H_{ij}\right)\;r^{j}\right]}_{\Sigma }=0,\; \end{array}$$

where $r^{j}$ is a unit radial vector. From Equations (3) and (24), we obtain the following:

(25) $$\begin{array}{c} {\left[T_{ij}\;r^{j}\right]}_{\Sigma }=0\;\;\;⟹\;\;\;{\left[p_{r}\right]}_{\Sigma }=0. \end{array}$$

Now, Equations (22), (23) and (25) describe the necessary and sufficient conditions for determining the arbitrary constants and parameters involved in the solution. Now by plugging λ(R) and ν(R) from Equations (13) and (16) into the conditions (22) and (23), and using condition (25), we obtain the expressions for the constants *C*, *M* and γ as the following:

$$\begin{array}{ccc} & & \;C=-\frac{1}{3\;\sqrt{\left(1+bR^{2}+cR^{4}\right)}}[\frac{\alpha \left(\nu_{1}+\nu_{2}\right)\left(-3-3\beta +4\alpha \gamma \right)\ln\left[b+4\alpha c-\nu_{5}+2cR^{2}\right]}{\nu_{5}} \end{array}$$

(26) $$\begin{array}{ccc} & & \;+\frac{1}{6}c\;\gamma \;R^{6}+\nu_{3}\;R^{2}+\nu_{4}\;R^{4}+\frac{3}{2}\;\beta \;\ln\left(1+bR^{2}+cR^{4}\right)], \end{array}$$

(27) $$\begin{array}{ccc} & & \;M=\frac{R^{4}\left(b+cR^{2}\right)\left[1+bR^{2}+cR^{4}+2\alpha \left(b+cR^{2}\right)\right]}{2{\left(1+bR^{2}+cR^{4}\right)}^{2}}, \end{array}$$

(28) $$\begin{array}{ccc} & & \;\gamma =-\frac{3\beta }{{\left(1+bR^{2}+cR^{4}\right)}^{3}}[b^{3}R^{4}+cR^{2}\left(3+8\alpha cR^{2}+4cR^{4}+c^{2}R^{8}\right)+b(2+12\alpha cR^{2}\\ & & \;+7cR^{4}+3c^{2}R^{8})+b^{2}\left\{4\alpha +3\left(R^{2}+cR^{6}\right)\right\}]. \end{array}$$

Now, these determined constants will be used to discuss the physical properties of our obtained solution. On the other hand, some of the free constants and parameters are fixed, based on the observational constraints from various pulsar measurements, and corresponding to their mass-radius ratio.

## 5. Physical Analysis of the Obtained Solution

In order to check the physical viability of the anisotropic solution, we perform some analytical calculations with different aspects, such as the positivity of central density ($\rho_{c}$), central pressures ($p_{rc}\;\mathrm{and}\;p_{tc})$ and the Zeldovich condition $\left(\frac{p_{rc}}{\rho_{c}}=\frac{p_{tc}}{\rho_{c}}\leq 1\right)$:

(29) $$\begin{array}{ccc} & & \rho_{c}=\frac{3\;b\;(1+2\;\alpha \;b)}{4\;\pi }>0,\;\;⟹\;\;b>0, \end{array}$$

(30) $$\begin{array}{ccc} & & p_{rc}=p_{tc}=\frac{3b\beta +6\alpha b^{2}\beta +\gamma }{4\;\pi }\geq 0\;\;⟹\;\beta \geq -\frac{\gamma }{6b+12\alpha b^{2}}, \end{array}$$

(31) $$\begin{array}{ccc} & & \frac{p_{rc}}{\rho_{c}}=\frac{p_{rc}}{\rho_{c}}=\frac{3b\beta +6\alpha b^{2}\gamma +\gamma }{3\;b\;(1+2\;\alpha \;b)}\leq 1\;\;⟹\;\;0<\beta \leq \frac{6b+12\alpha b^{2}-\gamma }{6b+12\alpha b^{2}},\;\;\;\;\; \end{array}$$

From Equations (29)–(31), we find the lower and upper bounds for β as the following:

(32) $$\begin{array}{c} -\frac{\gamma }{6b+12\alpha b^{2}}\leq \beta \leq \frac{6b+12\alpha b^{2}-\gamma }{6b+12\alpha b^{2}}. \end{array}$$

Here, we investigate the influence of the Gauss-Bonnet coupling constant α on physical parameters, such as pressure, density, anisotropy, mass-radius ratio, redshift, etc. To check this, we consider a particular pulsar PSR J1416-2230 [77] with mass $1.97M_{⊙}$ and radius 9.62km or $\frac{M_{0}}{R}=0.3$ in corresponding to α=0, i.e., pure GR case in 5D, where $M_{0}$ is assumed to be a total mass of the PSR J1416-2230 in the 5D GR scenario.

### 5.1. Regularity Conditions

**Figure 1:**
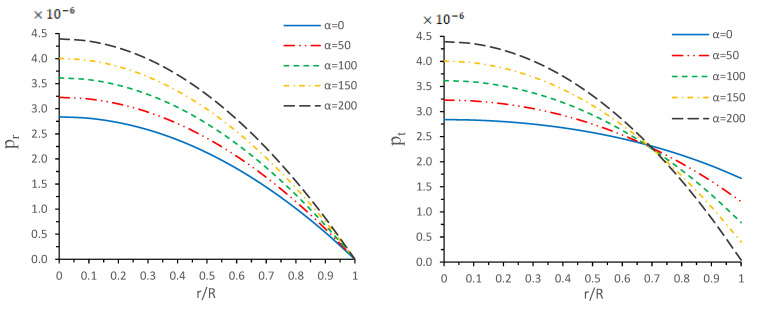
Variations of radial pressure ($p_{r}$) (**left panel**) and tangential pressure ($p_{t}$) (**right panel**) versus radial coordinate r/R for different values of α by taking $\frac{M_{0}}{R}=0.3$ with $b=0.0007\;{\mathrm{km}}^{-2}$, $c=2.3\times 10^{-7}\;{\mathrm{km}}^{-4}$, and β=0.33.

**Figure 2:**
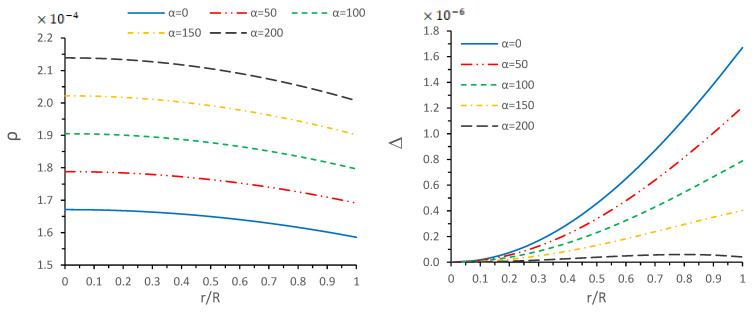
Variations of energy density (ρ) (**left panel**), and anisotropy (Δ) (**right panel**) versus radial coordinate r/R for different values of α by taking the same values of the constant as used in Figure 1.

From the above constraints for the energy density (ρ) and two different components of pressure (radial pressure ($p_{r}$) and transverse pressure $\left(p_{t}\right)$), we verified that the physical quantities, such as ρ, $p_{r}$ and $p_{t}$, are free from any kind of singularity. On the other hand, from Figure 1 and Figure 2, one verifies that all the physical quantities are maximal at the center and monotonically decreasing toward the surface. Additionally, these quantities, ρ, $p_{r}$ and $p_{t}$, are positive and finite at each point within the star. Moreover, the curves for the top left panel of Figure 1 show that the radial pressure vanishes at the surface of the star (r=R), which gives the radius of the star, but tangential pressure is not zero throughout the configuration; see Figure 1—right panel. Moreover, from Figure 2—left panel—we observe that the density at the core and surface increases when α increases, and the trend of energy density curves shows a monotonic decreasing behavior for each α. On the other hand, we also measure the pressures anisotropy inside the configuration. It is clear from Figure 2—right panel—that the Δ>0, i.e., $p_{t}>p_{r}$ increase monotonically throughout the star for α=0,50,100 and 150, while the anisotropy does not behave like the monotonic increasing behavior for large value of α since for α=200, the anisotropy starts increasing and attains its maximum value at r/R≈0.8, and starts decreasing after this value. Finally, we conclude that the anisotropic force will be repulsive due to Δ>0, but this would introduce the lessened effect to achieve the hydrostatic equilibrium for increasing values of α.

### 5.2. Causality

In addition to the above requirements, it is important to satisfy the physical acceptability conditions, such as the causality condition, which states that the squares of radial and tangential sound speeds for anisotropic matter should be less than unity throughout the model, i.e.,

(33) $$\begin{array}{c} v_{r}^{2}=\frac{dp_{r}}{d\rho }<1\;\;\;\mathrm{and}\;\;\;0<v_{t}^{2}=\frac{dp_{t}}{d\rho }<1, \end{array}$$

**Figure 3:**
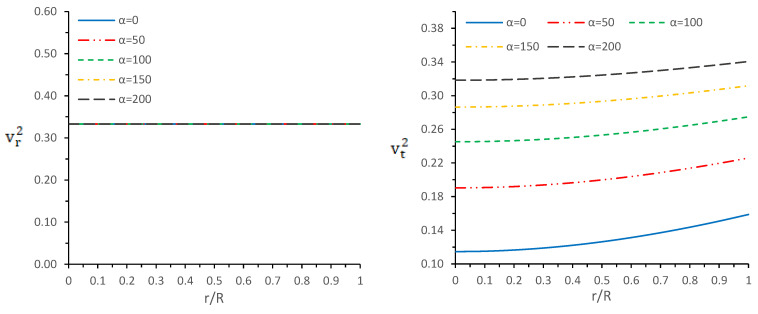
Variations of radial velocity ($v_{r}^{2}$) (**left panel**) and tangential velocity ($v_{t}^{2}$) (**right panel**) versus radial coordinate r/R for different values of α by taking $\frac{M_{0}}{R}=0.3$ with $b=0.0007\;{\mathrm{km}}^{-2}$, $c=2.3\times 10^{-7}\;{\mathrm{km}}^{-4}$, and β=0.33.

In other words, these conditions state that the velocity of sound must be less than the velocity of light. Since we have considered a linear EOS for radial pressure, then from Equation (13), we obtain $v_{r}^{2}=\beta$. Therefore, the radial velocity is constant throughout the star and solely depends on parameter β, while tangential velocity is not constant. To see the exact tread of these velocities, Figure 3 is plotted by taking β=1/3; we observe that the tangential velocity is also less than unity, but it shows monotonic increasing behavior when we move toward the surface. Finally, we can say that both velocities satisfy the causality conditions.

### 5.3. Stability of Anisotropic Compact Objects via Cracking

Herrera and his collaborators [38,78,79] proposed the concept of cracking for self-gravitating by taking a perfect fluid and anisotropic matter distributions. This concept was presented to define the variation of fluid distributions just after its departure from equilibrium in the presence of total non-vanishing radial forces with different signs. In this section, we will discuss the cracking approach to find potentially stable (and unstable) anisotropic matter configurations. For this purpose, we use Abreu’s criterion [80], which state that the pressure waves of the spherically symmetric star are split in two principal directions, namely the subliminal radial sound speed and subliminal tangential sound speed, and depending on their values at some particular points of the object, where the stability of the system is determined. This mechanism can be easily characterized in the following way:

(34) $$\frac{\delta \Delta }{\delta \rho }∼\frac{\delta \left(p_{t}-p_{r}\right)}{\delta \rho }∼\frac{\delta p_{t}}{\delta \rho }-\frac{\delta p_{r}}{\delta \rho }∼v_{t}^{2}-v_{r}^{2}.$$

since we have $0\leq v_{r}^{2}\leq 1$ and $0\leq v_{t}^{2}\leq 1$ from the causality condition, which leads to $0\leq |v_{t}^{2}-v_{r}^{2}|\leq 1$. Additionally, the potentially stable stellar structures should satisfy $\frac{\delta \Delta }{\delta \rho }<0$. Then, finally, this can be explicitly read as the following:

(35) $$\begin{array}{c} -1\leq v_{t}^{2}-v_{r}^{2}\leq 1=\left\{\begin{array}{cc} -1\leq v_{t}^{2}-v_{r}^{2}\leq 0\;\; & \mathrm{Potentially}\;\mathrm{stable}\;\\ 0<v_{t}^{2}-v_{r}^{2}\leq 1\;\; & \mathrm{Potentially}\;\mathrm{unstable} \end{array}\right\}.\;\;\;\; \end{array}$$

**Figure 4:**
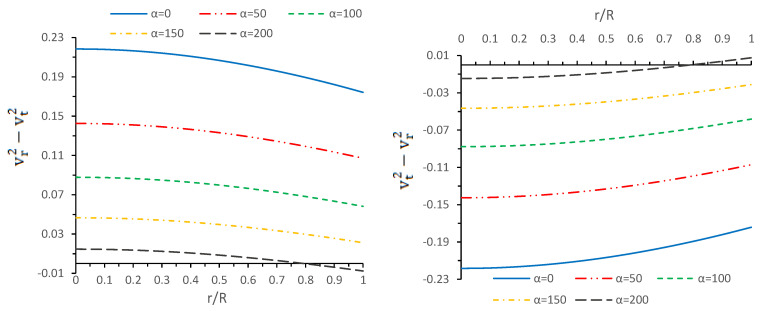
Variations of stability factors ($v_{r}^{2}-v_{t}^{2}$) (**left panel**) and ($v_{t}^{2}-v_{r}^{2}$) (**right panel**) versus radial coordinate r/R for different values of α by taking same values of the constant as used in Figure 3.

So in a nutshell, we can say that cracking instabilities lead to an unstable configuration if the subliminal tangential speed $v_{t}^{2}$ becomes greater than the subliminal radial speed $v_{r}^{2}$. So, by graphical representation of Figure 4, we observe that the radial speed of sound is greater than the tangential speed of sound throughout the star, i.e., $(v_{r}^{2}-v_{t}^{2})>0$, for α=0,50,100,150 while $(v_{r}^{2}-v_{t}^{2})<0$ for α=200 when r/R≥0.7, which implies that the system leads an unstable region for large values of Gauss-Bonnet coupling constant α.

### 5.4. The Stability Criterion and the Adiabatic Indices

In this section, we will discuss the stability analysis of the solution via the adiabatic index Γ. Chandrasekhar [81] proposed the method to study the dynamical stability based on the variational method. Based on this method [81,82], an important relation for the adiabatic index, Γ was derived as $\Gamma \equiv \left(1+\rho /p_{r}\right)\;{\left(\frac{dp_{r}}{d\rho }\right)}_{S}$, where $dp_{r}/d\rho$ is the speed of sound in units of speed of light and the subscript *S* indicates the derivation at constant entropy. However, this principle was modified in the presence of anisotropy and radiation (in the free-streaming) for discussing the dynamical instabilities of the models. In this connection, the new constraints on adiabatic index Γ were proposed by Moustakidis [83], who claimed the existence of a critical value for the adiabatic index, denoted by $\Gamma_{\mathrm{crit}}$, depending on the amplitude of the Lagrangian displacement from equilibrium and the compactness factor u=M/R [84] as follows:

(36) $$\Gamma_{\mathrm{crit}}=\frac{4}{3}+\frac{19}{21}\;u,$$

**Figure 5:**
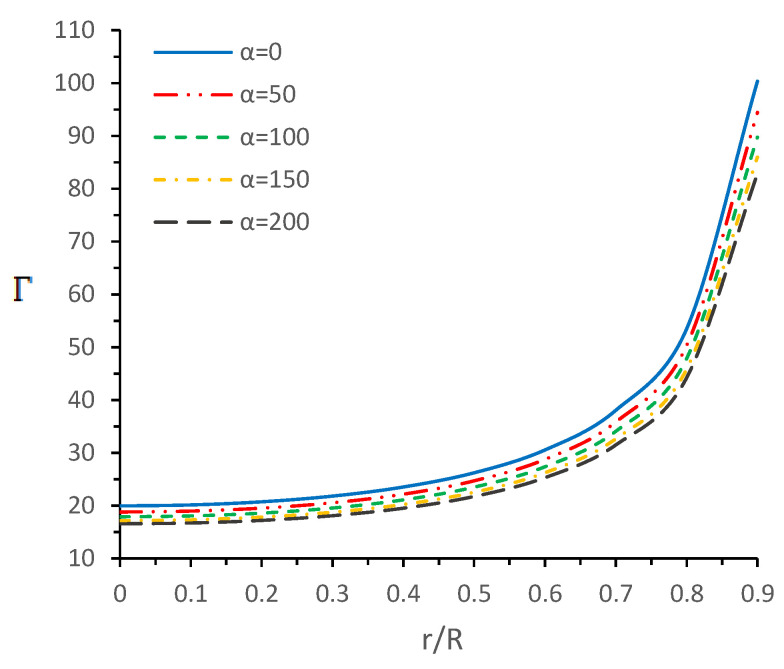
Variations of adiabatic index (Γ) versus radial coordinate r/R for different values of α by taking $\frac{M_{0}}{R}=0.3$ with $b=0.0007\;{\mathrm{km}}^{-2}$, $c=2.3\times 10^{-7}\;{\mathrm{km}}^{-4}$, and β=0.33.

The anisotropic solution describes a stable model if $\Gamma \geq \Gamma_{\mathrm{crit}}$. In the scenario of neutron star models, the value of adiabatic index Γ can lie between 2 and 4 [85]. Figure 5 shows the trend of the adiabatic index inside the stellar object, which increases monotonically toward the boundary. Additionally, numerical values of $\Gamma_{c}$ at r=0 and $\Gamma_{crit}$ are mentioned in the Table 1. It can be observed that the resulting $\Gamma_{c}>\Gamma_{\mathrm{crit}}$ for all taken α values, which shows that our model is stable against the radial adiabatic infinitesimal perturbations.

### 5.5. Hydrostatic Equilibrium via Modified TOV Equation

In this section, we will check the hydrostatic equilibrium of the system via the modified Tolman-Oppenheimer-Volkoff (TOV) equation under different forces. To analyze, we write the general form of the modified TOV equation as follows:

(37) $$\begin{array}{c} \frac{M_{G}\left(r\right)\left(\rho +p_{r}\right)}{r^{2}}e^{\frac{\lambda -\nu }{2}}+p_{r}^{\prime }+\frac{3}{r}\left(p_{r}-p_{t}\right)=0, \end{array}$$

where the effective gravitational mass $M_{G}\left(r\right)$ can be given as the following:

(38) $$\begin{array}{cc} & \;\;M_{G}\left(r\right)=r^{2}e^{\frac{\nu -\lambda }{2}}\nu^{\prime }. \end{array}$$

Thus, the above TOV equation can be divided into three different forces, as the gravitational force [$F_{g}=-\nu^{\prime }\;\left(\rho +p_{r}\right)$], hydrostatic-gradient [$F_{h}=-p_{r}^{\prime }$] and another force due to the anisotropic pressure [$F_{a}=\frac{3(p_{t}-p_{r})}{r}$]; then, the system is in an equilibrium position if the sum of all the forces is zero, i.e.,

(39) $$F_{g}+F_{h}+F_{a}=0,$$

**Figure 6:**
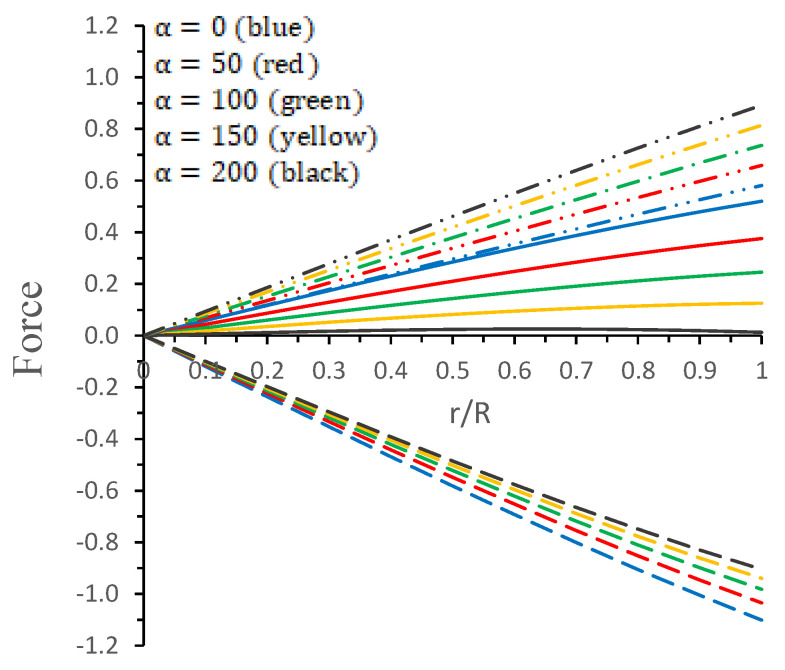
Variations of different forces, such as the hydrostatic force ($F_{h}$) (long dash dotted curve), anisotropic force ($F_{a}$) (long dash curve), and gravitational force ($F_{g}$) (solid curve) versus radial coordinate r/R for different values of α. We used the same values of constants as those used in Figure 5.

In Figure 6, the variation of the above different forces against the radial coordinate r/R for different values of the coupling constant α are presented. It is observed that the joint action of the repulsive forces, hydrostatic $F_{h}$ force and anisotropic $F_{a}$ force, is counterbalanced by the attractive gravitational force $F_{g}$ such that $F_{g}+F_{h}+F_{a}=0$, which shows that system is in hydrostatic equilibrium.

### 5.6. Mass-Radius Relationship

**Figure 7:**
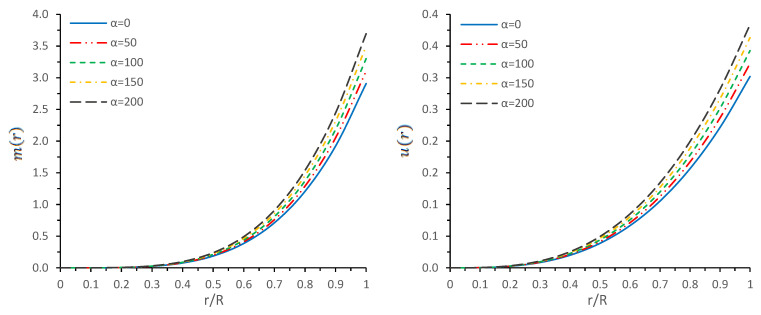
Variations of mass function (m(r)) and compactness (u=m(r)/r) versus radial coordinate r/R for the compact object for different values of α.

Since we successfully tested that our solution describes the physically viable stellar model, now we will proceed to discuss the most important physical parameters, such as surface redshift and mass-radius ratio or compactness (u=M/R). In general, the surface gravitational redshift $z_{s}$ of the compact object can be given by the following definition $z_{s}$ = $\frac{\lambda_{0}-\lambda_{e}}{\lambda_{e}}$, where $\lambda_{e}$ is the emitted wavelength of the non-rotating compact star models at the surface, while $\lambda_{0}$ represents an observed wavelength received at radial coordinate *r*. For our spacetime, the surface gravitational redshift $z_{s}$ can be obtained by the following formula:

(40) $$\begin{array}{c} z_{s}\left(R\right)=e^{-\nu (R)/2}-1=\frac{1}{\sqrt{1-2u(R)}}-1. \end{array}$$

where u(R)=M/R denotes a compactification factor of the stellar compact object. Buchdahl [86] and Straumann [87] had fixed an upper bound of the gravitational redshift for perfect fluid spheres, which is $z_{s}<$ 2. After a few years, this upper bound was further extended by Ivanov [88], who admitted the higher redshifts $z_{s}$ = 3.84 in the presence of anisotropic matter distribution. On the other hand, Buchdahl [86] proposed the upper bound limit of the compactness factor by assuming a positive monotonic decreasing energy density, i.e., dρ/dr≤0, toward the surface of the star, which is u≤4/9, where *M* and *R* denote the mass and radius of the object, respectively. Thus, the surface redshift of the compact object cannot be arbitrarily large. The numerical results are presented in Table 1. The variations of the mass functions (m(r)) and compactness function (u(r)) within the stellar object are shown by Figure 7. From this Figure 7, it is observed that both m(r) and u(r) are increasing towards the boundary and its value in magnitude increases with α.

### 5.7. Maximum Mass and Fitting of the Radius for Known Compact Objects via M−R Curve

**Figure 8:**
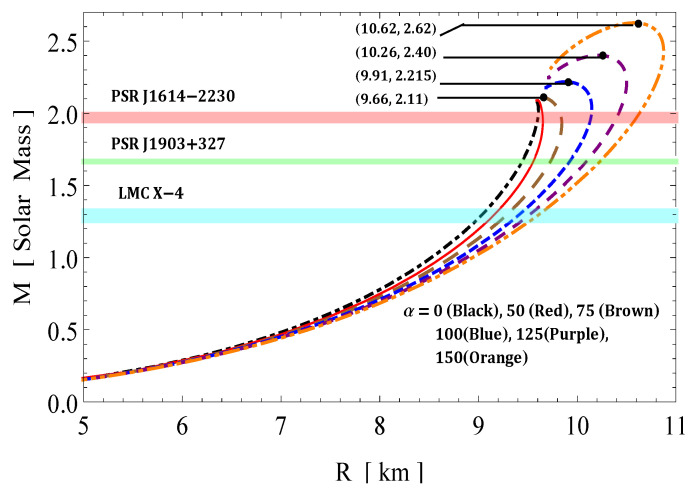
Variations of M−R curves for different values of α.

Figure 8 shows the M−R curves for different values of GB constant α. The maximum mass and corresponding radius for different values of α are as follows: (i) for α=0, (ii) for α=50, (iii) $M_{max}=2.11M_{⊙}$ with R=9.66km for α=75, (iv) $M_{max}=2.115M_{⊙}$ with R=9.91km for α=100, (v) $M_{max}=2.40M_{⊙}$ with R=10.26km for α=125, and (vi) $M_{max}=2.62M_{⊙}$ with R=10.62km for α=150. We have also fitted some known compact stars, such as PSR J1614-2230, PSR J1903+317, and LMC X-4, whose masses and predicted radii for different values of the GB constant α are given in the Table 2.

## 6. Concluding Remarks

In this paper, a new kind of anisotropic solution for a strange star model in the context of 5*D* Einstein-Gauss-Bonnet (EGB) gravity was studied. To this end, we used a well-behaved ansatz for the gravitational potential corresponding radial component of the spacetime together with a linear equation of the state $p_{r}=\beta \;\rho +\gamma$ (β and γ being constants). Thus, we obtained the other gravitational potential along with the key thermodynamical variables, such as radial and tangential pressures, energy density, anisotropy, etc. However, we obtained the constant parameters by matching the well-recognized Boulware-Deser solution at the boundary. One of our primary focuses were to validate our model with the existence of strange stars consistent with the observed data and how its physical properties vary with Gauss-Bonnet constant α; hence, we cited the particular pulsar PSR J1416-2230 having mass $1.97M_{⊙}$. For checking the viability of our findings, we further analyzed several key and important physical features of the stellar configuration both by graphical and analytical representation. We used the much known Boulware-Deser metric to define the exterior spacetime. Thus, by matching the interior and exterior spacetime at the boundary surface, we obtained the values of the arbitrary constants *C*, *M* and γ. After this, we used these constant parameters together with the mass-radius ratio $\frac{M}{R}=0.3$ corresponding to α=0 for studying the physical properties of the solution in EGB gravity. First, we examined the graphical representation of the pressure components (both radial and transverse), anisotropy factor, and energy density; see Figure 1 and Figure 2. We took note of the fact that the anisotropy vanishes at the center and gradually increases as we move toward the boundary. The nature of all the plots is physically viable. Furthermore, we checked the causality condition (Figure 3) and found out that $v_{r}^{2}$ and $v_{r}^{2}$ holds well the inequality of the causality condition, i.e., $0<v_{r}^{2}<1$ and $0<v_{t}^{2}<1$, which implies that our model behaves consistently with the causality condition. After that, we used the cracking concept and adiabatic index for visualizing the stability of the model. For this purpose, we plotted Figure 4 to test the cracking condition; we found that there is no cracking within the system, and the radial velocity of sound is greater than the tangential velocity throughout the model, making the model stable. However, the variation of the relativistic adiabatic index Γ is shown in Figure 5, and it tested our model’s stability against the radial adiabatic infinitesimal perturbations by showing that the relativistic adiabatic index (Γ) has to be greater than $\Gamma_{\mathrm{crit}}$ (see Table 1 for more details). In addition, we plotted the variation of the hydrostatic $F_{h}$, gravitational $F_{g}$ and anisotropic pressure $F_{a}$ forces in Figure 6, and we find that the model is in stable equilibrium. Finally, by considering the reference of the above well-measured observed mass for PSR J1416-2230 at α=0, we conclusively found that the maximum masses in our model is beyond 2 $M_{⊙}$ when α moves from 0 to 200. It is also evident that the compactness u=M/R is dependent on α. It is obvious that α=0 signifies the GR solution in 5D. So, if α is non zero, i.e., when EGB theory is being incorporated, then the compactness (u) is increasing when α increases, which shows that we obtain a more compact object in EGB gravity as compared to GR (see Figure 7). The obtained values of the physical parameters for several values of the GB coupling constant α are mentioned in Table 1. The M−R curves are also plotted in Figure 8 to find the maximum mass limit for different values of GB constant α. We fitted the radii for some known compact objects, PSR J1614-2230, PSR J1903+317, and LMC X-4, via the M−R curves, which are presented in Table 2.

## Figures and Tables

**Table 1:** The values of the physical parameters for different values of α by taking $\frac{M_{0}}{R}=0.3$ with $b=0.0007\;{\mathrm{km}}^{-2}$, $c=2.3\times 10^{-7}\;{\mathrm{km}}^{-4}$, and β=0.33. We denote $\Gamma_{c}$ as the central value of the adiabatic index Γ.

α	$M/M_{⊙}$	$\frac{M}{R}$	Surface	Central Density	Surface Density	Central Pressure	$\Gamma_{c}$	$\Gamma_{\mathrm{crit}}$	γ
(km^−2^)			Red-Shift ($z_{s}$)	($\rho_{c}$) in g/cm^3^	($\rho_{s}$) in g/cm^3^	($p_{c}$) in dyne/cm^2^			km^−2^
0	1.97	0.30	0.589	$2.255\times 10^{14}$	$2.140\times 10^{14}$	$3.450\times 10^{33}$	19.928	1.606	0.00099542
50	2.10	0.32	0.677	$2.413\times 10^{14}$	$2.282\times 10^{14}$	$3.922\times 10^{33}$	18.776	1.625	0.0010615
100	2.24	0.34	0.783	$2.571\times 10^{14}$	$2.424\times 10^{14}$	$4.394\times 10^{33}$	17.872	1.643	0.0011276
150	2.37	0.36	0.911	$2.729\times 10^{14}$	$2.567\times 10^{14}$	$4.866\times 10^{33}$	17.143	1.662	0.0011937
200	2.5	0.38	1.072	$2.887\times 10^{14}$	$2.709\times 10^{14}$	$5.339\times 10^{33}$	16.54	1.680	0.0012598

**Table 2:** The predicted radii for known compact stars, such as PSR J1614-2230, PSR J1903+317, and LMC X-4 with different values of GB constant α.

Objects	$\frac{M}{M_{⊙}}$	Predicted *R* km					
		α					
		0	50	75	100	125	150
PSR J1614-2230	1.97 ± 0.04	$9.60_{-0.013}^{+0.05}$	$9.646_{-0.006}^{+0.003}$	$9.84_{-0.016}^{+0.002}$	$10.13_{-0.01}^{+0.01}$	$10.40_{-0.03}^{+0.02}$	$10.60_{-0.04}^{+0.03}$
PSR J1903+327	1.667 ± 0.021	$9.446_{-0.021}^{+0.016}$	$9.557_{-0.01}^{+0.016}$	$9.73_{-0.016}^{+0.017}$	$9.92_{-0.016}^{+0.026}$	$10.09_{-0.03}^{+0.03}$	$10.23_{-0.02}^{+0.03}$
LMC X-4	1.29 ± 0.05	$9.025_{-0.074}^{+0.065}$	$9.146_{-0.07}^{+0.069}$	$9.27_{-0.074}^{+0.074}$	$9.395_{-0.083}^{+0.079}$	$9.50_{-0.082}^{+0.089}$	$9.594_{-0.097}^{+0.093}$

## Data Availability

This manuscript has no associated data.

## Appendix A. Complexity for Self-Gravitating Fluid Distributions under the Static Spherically Symmetric Spacetime

The concept of “complexity” is often very vague and subjective. Quantifying the term “complexity” for a particular context is an interesting challenge. In a similar thought process, another specific concept of “disequilibrium” was proposed [89], which signifies the deviation from a uniform distribution of the accessible states of the system. Clearly, for a perfect crystal, “disequilibrium” is maximal, while for an ideal gas, it is zero. Now, a perfect crystal has maximum ordering and the ideal gas has maximum “information”, as we can find the system in many states having the same probability. By combining the concept of “disequilibrium” with “information”, this leads to the definition of complexity in mathematical form [89]. This definition of complexity, as developed by Lopez-Ruiz and their collaborators [89,90], was proposed for self-gravitating systems as well [91,92,93,94,95].

Later, Herrera [96] proposed a new definition for the complexity for static, spherically symmetric, and self-gravitating systems by defining a quantity referred to as the complexity factor. He considered the framework of Einstein’s general relativity theory in this context. The essence behind this definition of complexity is that it takes into account deviation in the value of the Tolman mass from the value for a zero-complexity system. For testing this approach, he tested a homogeneous isotropic fluid distribution and found its complexity factor to be zero. He proposed a quantity $Y_{TF}$ as the complexity factor which has the following mathematical form:

(A1) $$\begin{array}{c} Y_{TF}=8\pi \Delta -\frac{4\pi }{r^{3}}\int_{0}^{r}{\tilde{r}}^{3}\rho \;d\tilde{r} \end{array}$$

It must be noted that the complexity factor $Y_{TF}$, which is basically a structure scalar, takes into account the influence of the density inhomogeneity, and pressure anisotropy on the Tolman mass. If the electric charge is included, then the contribution of the electric charge is included in the corresponding $Y_{TF}$. Additionally, the definition of the quantity $Y_{TF}$ is such that it not only vanishes for the homogeneous isotropic fluid, but also, both its terms as seen in Equation (A1) vanish subsequently. In our case, the complexity factor is the following:

(A2) $$\begin{array}{c} Y_{TF}=8\pi \Delta -\frac{6\pi [2\alpha -\left(4\alpha +r^{2}\right)\left(1+br^{2}+cr^{4}\right)+{\left(r+br^{3}+cr^{5}\right)}^{2}]}{r^{3}{\left(1+br^{2}+cr^{4}\right)}^{2}} \end{array}$$

where $\Delta =p_{t}-pr$ can be obtained, using Equations (18) and (19).

The expression for used coefficients $p_{t1}$ and $p_{t2}$ in Equation (19) are as follows:

(A3) $$\begin{array}{ccc} & & \;p_{t1}=b^{6}r^{10}{\left(3+3\beta +\gamma r^{2}\right)}^{2}+\gamma^{2}r^{2}{\left(1+cr^{4}\right)}^{6}+3\gamma {\left(1+cr^{4}\right)}^{3}(4acr^{2}\left(3+c\left(5+4\beta \right)r^{4}\right)\\ & & \;+\left(1+2c\left(1+\beta \right)r^{4}\right)\left(3+4cr^{4}+c^{2}r^{8}\right))+3b^{5}r^{6}(4a\left(3+6\beta^{2}+4\gamma r^{2}+2\beta \left(6+\gamma r^{2}\right)\right)\\ & & \;+r^{2}(18\beta^{2}\left(1+cr^{4}\right)+12\beta \left(3+\gamma r^{2}\right)\left(1+cr^{4}\right)+3\left(5+6cr^{4}\right)+2\gamma r^{2}\left(7+6cr^{4}\right)\\ & & \;+2\gamma^{2}\left(r^{4}+cr^{8}\right)))+9cr^{2}(cr^{4}{\left(1+cr^{4}\right)}^{2}\left(3+8acr^{2}+4cr^{4}+c^{2}r^{8}\right)+c\beta^{2}r^{4}(3+8acr^{2}\\ & & \;+4cr^{4}+c^{2}r^{8}{)}^{2}+\beta (-32a^{2}c^{2}r^{4}\left(-7+3cr^{4}\right)+{\left(1+cr^{4}\right)}^{2}\left(15+12cr^{4}+11c^{2}r^{8}+2c^{3}r^{12}\right)\\ & & \;+4acr^{2}\left(29+33cr^{4}+11c^{2}r^{8}+7c^{3}r^{12}\right)))+3b^{4}r^{2}(48a^{2}\left(-2+\beta \right)\beta +12ar^{2}(2+6cr^{4}\\ & & \;+6\beta^{2}\left(1+2cr^{4}\right)+\gamma r^{2}\left(5+7cr^{4}\right)+\beta \left(9+23cr^{4}+2\gamma \left(r^{2}+2cr^{6}\right)\right))+r^{4}(27+78cr^{4}\\ & & \;+45c^{2}r^{8}+5r^{4}{\left(\gamma +c\gamma r^{4}\right)}^{2}+\gamma r^{2}\left(39+71cr^{4}+30c^{2}r^{8}\right)+\beta^{2}\left(39+96cr^{4}+45c^{2}r^{8}\right)\\ & & \;+\beta \left(9\left(9+21cr^{4}+10c^{2}r^{8}\right)+2\gamma r^{2}\left(14+31cr^{4}+15c^{2}r^{8}\right)\right)))+b^{3}(432a^{2}\beta \left(1+c\left(2\beta -3\right)r^{4}\right)\\ & & \;+12ar^{2}(3+30cr^{4}+42c^{2}r^{8}+12\beta^{2}\left(1+8cr^{4}+7c^{2}r^{8}\right)+\gamma r^{2}\left(21+63cr^{4}+44c^{2}r^{8}\right)\\ & & \;+2\beta \left(21+66cr^{4}+78c^{2}r^{8}+\gamma r^{2}\left(3+15cr^{4}+14c^{2}r^{8}\right)\right))+r^{4}(20\gamma^{2}r^{4}{\left(1+cr^{4}\right)}^{3}\\ & & \;+36\beta^{2}\left(3+15cr^{4}+17c^{2}r^{8}+5c^{3}r^{12}\right)+24\gamma r^{2}\left(7+20cr^{4}+18c^{2}r^{8}+5c^{3}r^{12}\right)+9(7\\ & & \;+39cr^{4}+54c^{2}r^{8}+20c^{3}r^{12})+12\beta (24+90cr^{4}+99c^{2}r^{8}+30c^{3}r^{12}+2\gamma r^{2}(4+15cr^{4}\\ & & \;+16c^{2}r^{8}+5c^{3}r^{12}))))+3b(6c\beta^{2}r^{4}(6+29cr^{4}+96a^{2}c^{2}r^{4}+39c^{2}r^{8}+19c^{3}r^{12}+3c^{4}r^{16}\\ & & \;+4acr^{2}\left(13+26cr^{4}+9c^{2}r^{8}\right))+\left(1+cr^{4}\right)(r^{2}\left(1+cr^{4}\right)(2\gamma^{2}r^{2}{\left(1+cr^{4}\right)}^{3}+3cr^{2}(5+17cr^{4}\\ & & \;+6c^{2}r^{8})+2\gamma \left(9+28cr^{4}+25c^{2}r^{8}+6c^{3}r^{12}\right))+12a(c^{2}r^{6}\left(5+9cr^{4}\right)+\gamma (1+8cr^{4}+15c^{2}r^{8}\\ & & \;+8c^{3}r^{12})))+2\beta (24a^{2}c^{2}r^{4}\left(29-17cr^{4}\right)+12acr^{2}(\gamma \left(1+3cr^{4}\right){\left(r+cr^{5}\right)}^{2}+2(9+21cr^{4}\\ & & \;+12c^{2}r^{8}+8c^{3}r^{12}))+\left(1+cr^{4}\right)(2\gamma {\left(r+cr^{5}\right)}^{2}\left(1+8cr^{4}+3c^{2}r^{8}\right)+9(1+10cr^{4}+13c^{2}r^{8}\\ & & \;+10c^{3}r^{12}+2c^{4}r^{16})))), \end{array}$$

(A4) $$\begin{array}{ccc} & & \;p_{t2}=3b^{2}(48a^{2}c\beta r^{2}\left(18+c\left(-18+13\beta \right)r^{4}\right)+4a(6c\beta^{2}r^{4}\left(9+31cr^{4}+16c^{2}r^{8}\right)+r^{2}(6cr^{2}(2\\ & & \;+9cr^{4}+8c^{2}r^{8})+\gamma \left(13+66cr^{4}+99c^{2}r^{8}+46c^{3}r^{12}\right))+\beta (3(9+65cr^{4}+72c^{2}r^{8}\\ & & \;+58c^{3}r^{12})+2\gamma \left(r^{2}+12cr^{6}+27c^{2}r^{10}+16c^{3}r^{14}\right)))+r^{2}(3\beta^{2}(4+46cr^{4}+103c^{2}r^{8}\\ & & \;+72c^{3}r^{1}2+15c^{4}r^{16})+\left(1+cr^{4}\right)(6+66cr^{4}+123c^{2}r^{8}+45c^{3}r^{12}+5\gamma^{2}r^{4}{\left(1+cr^{4}\right)}^{3}\\ & & \;+2\gamma r^{2}\left(22+65cr^{4}+58c^{2}r^{8}+15c^{3}r^{12}\right))+3\beta (2\gamma {\left(r+cr^{5}\right)}^{2}\left(3+12cr^{4}+5c^{2}r^{8}\right)+3(7\\ & & \;+40cr^{4}+65c^{2}r^{8}+46c^{3}r^{12}+10c^{4}r^{16})))). \end{array}$$
